# Characteristics of serum metabolites in sporadic amyotrophic lateral sclerosis patients based on gas chromatography-mass spectrometry

**DOI:** 10.1038/s41598-021-00312-8

**Published:** 2021-10-21

**Authors:** Rui Jia, Qiaoyi Chen, Qingqing Zhou, Ronghua Zhang, Jiaoting Jin, Fangfang Hu, Xiao Liu, Xing Qin, Li Kang, Songzhen Zhao, Yonghui Dang, Jingxia Dang

**Affiliations:** 1 Department of Neurology, The First Affiliated Hospital, Xi’an Jiaotong University, 277 Western Yanta Rd, Xi’an, 710061 China; 2 Department of Cell Biology and Genetics, Xian Jiaotong University Health Science Center, Xi’an, China; 3 Key Laboratory of Environment and Genes Related to Diseases of the Education Ministry, Key Laboratory of the Health Ministry for Forensic Medicine, College of Medicine and Forensics, Xi’an Jiaotong University Health Science Center, Xi’an, China

**Keywords:** Diseases of the nervous system, Metabolomics

## Abstract

To identify differential metabolites and metabolic pathways and provide guidance for the novel biomarkers for diagnosis and prognosis of amyotrophic lateral sclerosis (ALS). ALS patients and people without nervous diseases were recruited. Metabolomic analysis was performed using gas chromatography-mass spectrometry (GC/MS). The orthogonal projections to latent structures discriminant analysis (OPLS-DA) were used to identify differential metabolites. Kyoto Encyclopedia of Genes and Genomes and MetaboAnalyst were used to identify metabolic pathways. 75 metabolites were detected and aligned. The OPLS-DA showed the metabolomic profile of ALS patients and those in the fast-progression and slow-progression ALS groups differed from that of CTRL (*p* < 0.05). The levels of maltose, glyceric acid, lactic acid, beta-alanine, phosphoric acid, glutamic acid, ethanolamine and glycine in ALS were significantly higher, while 2,4,6-tri-tert-butylbenzenethiol was lower. Glycine, serine and threonine metabolism, D-glutamine and D-glutamate metabolism, alanine, aspartate, and glutamate metabolism, beta-alanine metabolism, and pyruvate metabolism were significantly altered metabolic pathways in ALS. ROC was used to discriminate ALS from CTRL with an AUC of 0.898 (*p* < 0.001) using 2,4,6-tri-tert-butylbenzenethiol, beta-alanine, glycine, and ethanolamine. The serum metabolites and metabolic pathways in ALS patients are significantly altered compared with CTRL. These findings may contribute to the early diagnosis of ALS.

## Introduction

Amyotrophic lateral sclerosis (ALS) is a complicated neurodegenerative disease characterized by the rapid, progressive loss of motor neurons in the brain and spinal cord^1^. It has garnered increasing attention since the Ice Bucket Challenge in 2014^2^. The diagnosis of ALS mainly depends on the manifestation of neurological deficits (positive signs of injury in the upper and lower motor neurons) and electromyography examination (EMG) results. The mean delay time from symptom onset to diagnosis is more than 12 months^3^. ALS patients exhibit different rates of progression after onset and clinical heterogeneity. The average survival time of ALS patients is 2–5 years after diagnosis^4,5^. Respiratory failure caused by respiratory muscle paralysis is the main cause of ALS-related death^6^. However, the pathogenesis of ALS is still unclear^7^. Riluzole is approved drugs for ALS, but with limited efficacy^8,9^. Therefore, there is an urgent need to identify novel biomarkers for the diagnosis and treatment of ALS.

Approximately 5–10% of ALS is familial (FALS) with a Mendelian pattern of inheritance^4,10^. Since the discovery of the first ALS-related gene *superoxide dismutase 1* in 1993^11^, more than 30 genes have been identified in FALS^12^. Although more than 120 genetic variants are associated with ALS^3^, genetic etiology has only been determined in 60% and 11% of FALS and sporadic ALS cases, respectively^13^. Epigenetic studies indicate potential links between environmental factors and pathogenic genetic alterations mediated by DNA methylation and non-coding RNA, among others^3,14,15^. It has been proposed that environmental factors promote the onset and development of ALS by regulating gene expression^16,17^.

Endogenous small-molecular metabolites (< 1 kDa) are considered the end products of gene-environment interactions^18^ and are closely related to genomics, transcriptomics, proteomics, and the cellular environment^19^. Metabolomics is a postgenomic approach that measures small-molecule metabolites in living systems, providing essential information on dynamic metabolic responses of endogenous factors^20^ during disease progression^21^. Serum/plasma metabolomics is an easily accessible tool that has been widely used to explore metabolic pathways in neurodegenerative diseases^19,22^.

A state of hypermetabolism, mainly of muscular origin, was observed in transgenic ALS mice, suggesting that hypermetabolism may increase the vulnerability of motor neurons^23^. Analyses of 101 metabolites associated with Alzheimer’s disease, Parkinson’s disease, and ALS have shown that uric acid, choline, creatinine, L-glutamine, alanine, creatinine, and N-acetyl-L-aspartate are common metabolites in these diseases^18^.

In this study, we identified differential metabolites and metabolic pathways associated with ALS using non-target gas chromatography-mass spectrometry (GC/MS). These findings may contribute to a better understanding of the characteristics of serum metabolites in ALS patients.

## Results

### Patients’ characteristics

We analyzed the serum samples obtained from ALS patients (n = 23) and healthy subjects (n = 25) using untargeted GC/MS metabolomics. The ALS and CTRL groups were age- and gender-matched (*p* > 0.05). Compared with the CTRL group, ALS patients had lower serum levels of creatinine and albumin, and a higher level of creatine kinase (*p* < 0.05). To identify the differentially expressed serum metabolites in ALS patients with different rates of progression, the ALSFRS-r scores of all patients were recorded at their initial visit. According to the median progression rate (0.772)^24^, ALS patients were categorized into two groups: fast-progression (Δr ≥ 0.772, n = 12) and slow-progression (Δr < 0.772, n = 11) groups. The demographic characteristics, laboratory examination results, ALSFRS-r scores, and treatment of the two groups are shown in Table 1.

**Table 1:** Demographic characteristics of ALS patients and healthy subjects.

	ALS	Fast-Progression ALS	Slow-Progression ALS	CTRL
Age (mean ± SD)	52.5 ± 1.7	52.6 ± 7.3	52.1 ± 9.0	52.4 ± 2.1
Gender (M/F)	12/11	7/5	5/6	12/13
BMI (kg/m^2^, mean ± SD)	22.2 ± 0.6	22.0 ± 2.7	21.7 ± 3.3	23.4 ± 0.6
Forced vital capacity (FVC, mean ± SD)	78.3 ± 22.1	73.0 ± 23.5	88.5 ± 13.3	–
Bulbar onset (%)	4 (18.2)	3 (13.6)	1 (4.6)	–
Months from onset to diagnosis (mean ± SD)	10.3 ± 5.3	7.5 ± 2.4	13.7 ± 5.4	–
Riluzole for more than 6 months (%)	12 (54.5)	5 (20.0)	7 (28.0)	–
ALSFRS-r scores	38.4 ± 6.2	36.7 ± 5.5	41.9 ± 2.5	–
LDL (mmol/L, mean ± SD)	2.6 ± 0.1	2.8 ± 0.7	2.5 ± 0.6	2.4 ± 0.1
TG (mmol/L, mean ± SD)	1.3 ± 0.2	1.2 ± 0.9	1.4 ± 1.0	1.3 ± 0.1
TCHO (mmol/L, mean ± SD)	4.3 ± 0.1	4.6 ± 0.7	4.1 ± 0.7	4.1 ± 0.1
Serum Creatinine (μmol/L, mean ± SD)	50.3 ± 2.1^a^	51.9 ± 10.9	48.6 ± 7.6^c^	59.3 ± 2.1
Serum uric acid (μmol/L, mean ± SD)	275.9 ± 16.2	287.7 ± 91.3	274.2 ± 71.1	295.1 ± 13.7
Serum urea nitrogen (mmol/L, mean ± SD)	5.4 ± 0.4	5.1 ± 2.4	5.9 ± 2.9	5.1 ± 0.3
Serum albumin(g/L, mean ± SD)	40.9 ± 0.5^a^	41.2 ± 2.4^b^	41.0 ± 2.6^c^	44.1 ± 0.6
Serum creatine kinase (U/L, mean ± SD)	170.0 ± 23.5^a^	146.2 ± 73.4^b^	207.6 ± 154.9^c^	98.5 ± 11.5

^a^ALS versus CTRL, *t*-test, *p* < 0.05.

^b^Fast-progression ALS versus CTRL, *t*-test, *p* < 0.05.

^c^Slow-progression ALS versus CTRL, *t*-test, *p* < 0.05.

### Serum metabolic profiles of ALS patients and healthy subjects

**Figure 1:**
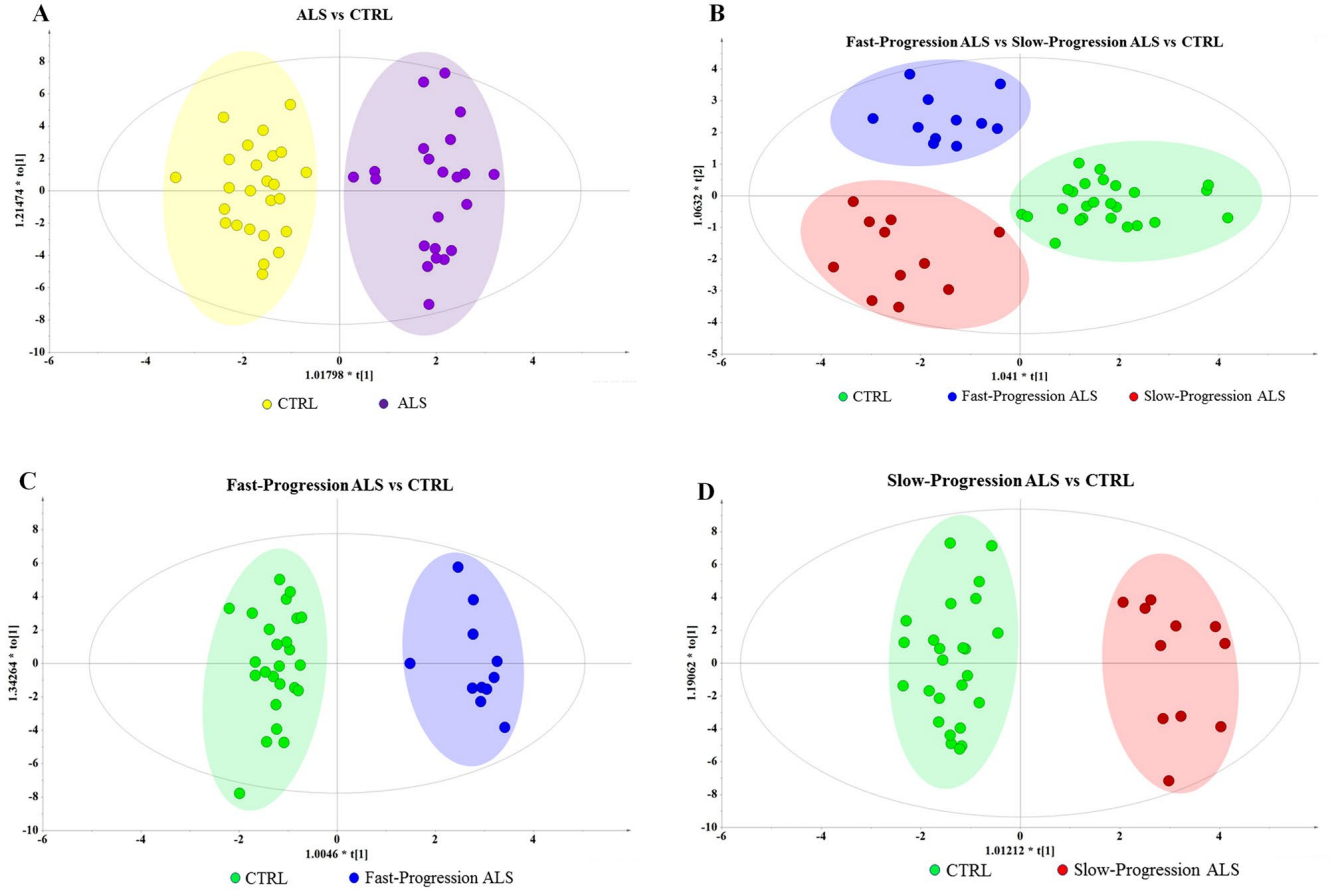
OPLS-DA score plots of ALS patients versus healthy subjects. OPLS-DA analysis shows a clear separation in (**A**) ALS versus CTRL, (**B**) fast-progression ALS versus slow-progression ALS versus CTRL, (**C**) fast-progression ALS versus CTRL, and (**D**) slow-progression ALS versus CTRL.

A total of 75 serum metabolites were detected and aligned after raw data processing including 25 amino acids (33%), 17 organic acids (23%), 9 fatty acids (12%), 7 sugars (9%), 6 polyols (8%), 3 phosphoric acids (4%), 2 amines (3%), and 6 others (8%). The OPLS-DA score plot was used to analyze serum metabolite changes and identify differential metabolites. The OPLS-DA score plots showed clear discrimination in ALS versus CTRL (cumulative R^2^Y = 0.901, Q^2^ = 0.602; Fig. 1A), fast-progression ALS versus slow-progression ALS versus CTRL (R^2^Y = 0.800, Q^2^ = 0.414; Fig. 1B), fast-progression ALS versus CTRL (R^2^Y = 0.953, Q^2^ = 0.676; Fig. 1C), and slow-progression ALS versus CTRL (R^2^Y = 0.937, Q^2^ = 0.56l; Fig. 1D).

### Analysis of differential serum metabolites and metabolic pathways

**Figure 2:**
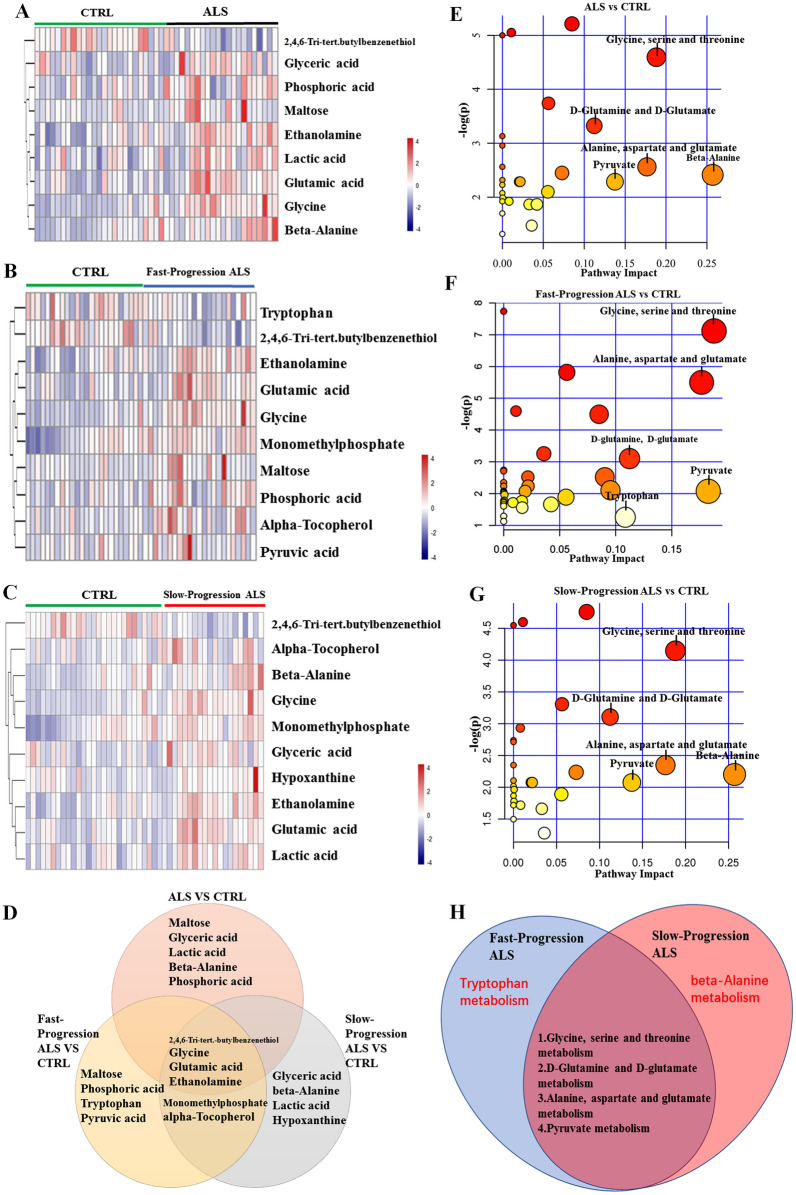
Heatmap visualization of differential serum metabolites and metabolic pathways. (**A**–**C**) Heatmap visualization of (**A**) ALS versus CTRL, (**B**) Fast-progression ALS versus CTRL, and (**C**) Slow-progression ALS versus CTRL. (**D**) Differential metabolites in the three comparison groups. (**E**–**G**) The altered metabolic pathways in the (**E**) ALS, (**F**) fast-progression ALS, and (**G**) slow-progression ALS groups compared with the CTRL group. (**H**) Differential metabolic pathways in the fast- and slow-progression ALS groups.

**Figure 3:**
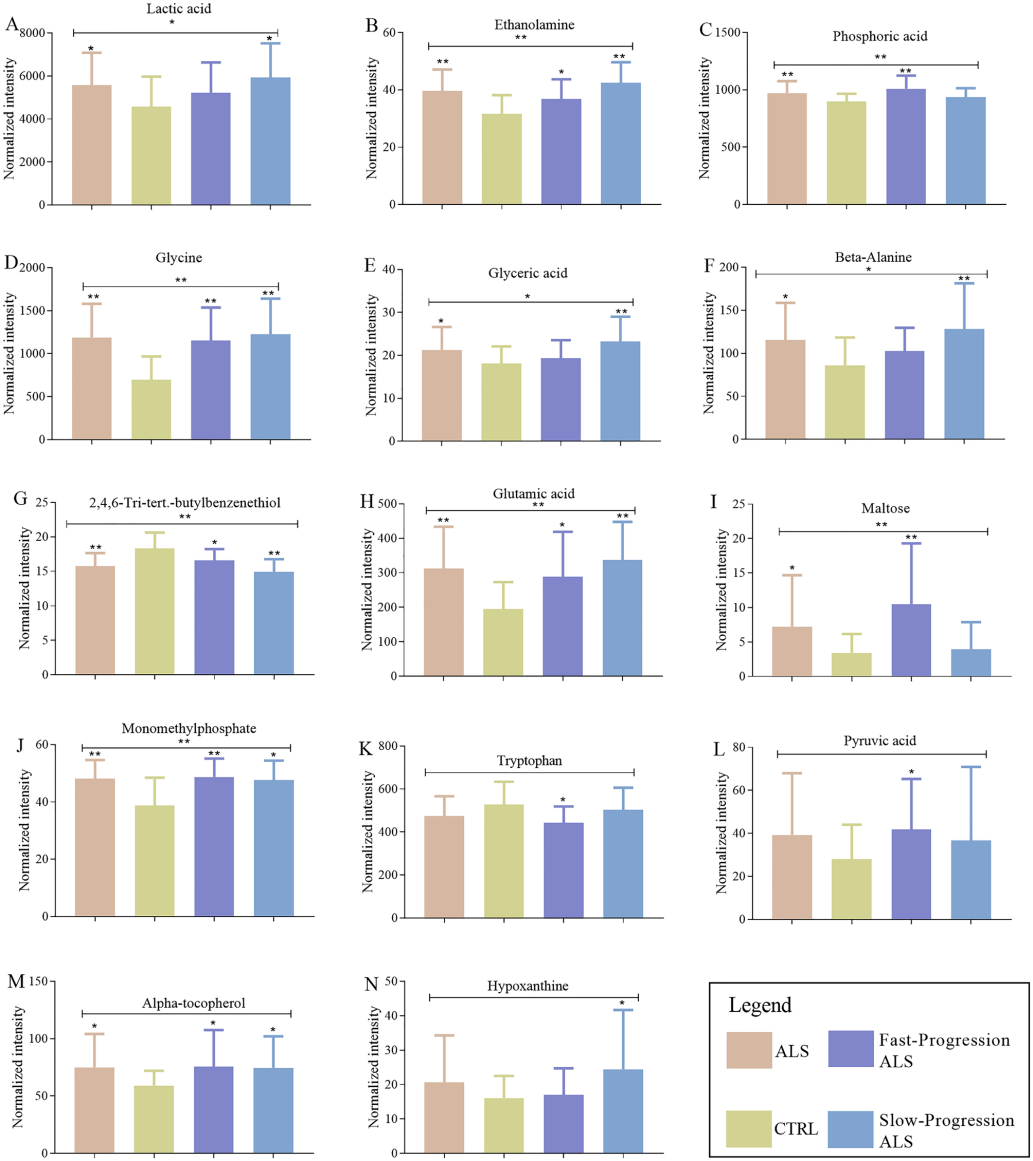
The analysis of each differential metabolites among ALS, ALS subgroups and CTRL. (**A**–**N**) The AVONVA analysis was used to compare each metabolite among ALS, CTRL, Fast-progression ALS and Slow-progression ALS; The Student’s *t*-test was used to compare each metabolite between the ALS and CTRL, Fast-progression ALS and CTRL, Slow-progression ALS and CTRL respectively. *p < 0.05; **p < 0.01.

Metabolites with a variable importance in the projection (VIP) > 1.0 and *p* < 0.05 in one-way analysis of variance were considered differential metabolites^25^ and are summarized in Table 2. Heatmaps of the differential metabolic profiles were generated based on hierarchical clustering analysis using the pheatmap package in R software. Compared with healthy subjects, ALS patients showed significantly higher levels of maltose, glyceric acid, lactic acid, beta-alanine, phosphoric acid, glutamic acid, ethanolamine, glycine, and a lower level of 2,4,6-tri-tert-butylbenzenethiol in the serum (Fig. 2A). The fast-progression ALS group showed significantly higher concentrations of maltose, glycine, phosphoric acid, monomethyl phosphate, glutamic acid, alpha-tocopherol, ethanolamine, and pyruvic acid, but lower levels of tryptophan and 2,4,6-tri-tert-butylbenzenethiol compared with the CTRL group (Fig. 2B). The serum concentrations of glycine, ethanolamine, glutamic acid, glyceric acid, beta-alanine, monomethyl phosphate, lactic acid, alpha-tocopherol, and hypoxanthine in the slow-progression ALS group were significantly higher, whereas the level of 2,4,6-tri-tert-butylbenzenethiol was lower compared with the CTRL group (Fig. 2C). By comparing fast- and slow-progression ALS patients with the CTRL group, we found that 2,4,6-tri-tert-butylbenzenethiol, glycine, glutamic acid, ethanolamine, monomethyl phosphate, and alpha-tocopherol were common differential metabolites in both subgroups. Maltose, phosphoric acid, tryptophan, and pyruvic acid were identified as differential metabolites only in the fast-progression ALS group, whereas glyceric acid, beta-alanine, lactic acid, and hypoxanthine were differential metabolites in the slow-progression ALS group (Fig. 2D). We also analysis the differential metabolites among ALS, ALS subgroups and CTRL by ANOVA, the results were shown in Fig. 3.

**Table 2:** Differential metabolites in ALS patients versus healthy subjects.

Metabolites	Mass-to-charge ratio	Retention time (min)	VIP value	Fold change	*p* value	q-value
**ALS versus CTRL**						
Glycine	174.1	10.148	2.133	0.584	< 0.001	< 0.001
Monomethyl phosphate	241.000	8.281	2.116	0.804	< 0.001	0.006
2,4,6-Tri-tert-butylbenzenethiol	263.100	13.202	1.837	1.163	< 0.001	0.005
Ethanolamine	174.072	9.575	1.835	0.796	< 0.001	0.005
Glutamic acid	246.100	14.117	1.773	0.624	< 0.001	0.005
Phosphoric acid	299.074	9.715	1.658	0.925	0.006	0.073
Beta-alanine	174.094	11.747	1.528	0.745	0.011	0.114
Maltose	361.075	24.220	1.449	0.470	0.021	0.164
Lactic acid	117.022	6.452	1.371	0.821	0.024	0.164
Alpha-tocopherol	237.094	27.392	1.249	0.788	0.017	0.161
Glyceric acid	189.016	10.506	1.141	0.850	0.024	0.164
**Fast-progression ALS versus CTRL**						
Glycine	174.100	10.100	2.632	1.660	< 0.001	0.019
Maltose	361.200	24.200	2.456	3.090	< 0.001	0.020
Phosphoric acid	299.100	9.700	2.442	1.120	< 0.001	0.020
Monomethyl phosphate	241.000	8.281	2.151	1.260	0.004	0.072
Glutamic acid	246.100	14.100	1.911	1.480	0.011	0.170
Tryptophan	202.098	3.368	1.714	0.842	0.025	0.307
2,4,6-Tri-tert-butylbenzenethiol	263.160	13.200	1.645	0.906	0.031	0.312
Alpha-tocopherol	237.094	3.952	1.629	1.280	0.033	0.312
Ethanolamine	174.100	9.600	1.592	1.170	0.038	0.315
Pyruvic acid	173.991	2.900	1.518	1.490	0.048	0.331
**Slow-progression ALS versus CTRL**						
Glycine	174.100	10.100	2.576	1.760	< 0.001	0.002
Ethanolamine	174.072	9.575	2.531	1.350	< 0.001	0.002
Glutamic acid	246.100	14.100	2.523	1.730	< 0.001	0.002
2,4,6-Tri-tert-butylbenzenethiol	263.100	13.202	2.495	0.814	< 0.001	0.002
Glyceric acid	189.016	10.506	1.968	1.280	0.004	0.057
Beta-alanine	174.094	11.747	1.889	1.490	0.006	0.071
Monomethyl phosphate	241.000	8.281	1.769	1.230	0.01	0.109
Lactic acid	117.022	6.452	1.68	1.300	0.015	0.142
Alpha-tocopherol	237.094	3.952	1.542	1.260	0.027	0.224
Hypoxanthine	265.000	16.181	1.435	1.520	0.04	0.303

Next, we analyzed the differential metabolites and altered metabolic pathways in ALS using the KEGG and MetPA databases. Relative-betweenness centrality was used to analyze pathway topology. Glycine, serine, and threonine metabolism, D-glutamine and D-glutamate metabolism, alanine, aspartate, and glutamate metabolism, beta-alanine metabolism, and pyruvate metabolism pathways were significantly altered between ALS patients and healthy subjects (Fig. 2E). The significantly altered pathways in the fast-progression ALS group were glycine, serine and threonine metabolism, alanine, aspartate, and glutamate metabolism, D-glutamine and D-glutamate metabolism, pyruvate metabolism, and tryptophan metabolism (Fig. 2F). The glycine, serine and threonine metabolism, alanine, aspartate, and glutamate metabolism, D-glutamine and D-glutamate metabolism, pyruvate metabolism, and beta-alanine metabolism were the significantly altered pathways between the slow-progression ALS and CTRL groups (Fig. 2G). Compared with the CTRL group, the tryptophan metabolism pathway was significantly enriched in the fast-progression ALS group, whereas the beta-alanine pathway was only significantly altered in the slow-progression ALS group (Fig. 2H).

### Receiver operating characteristic analysis of differential metabolites in different ALS groups

**Figure 4:**
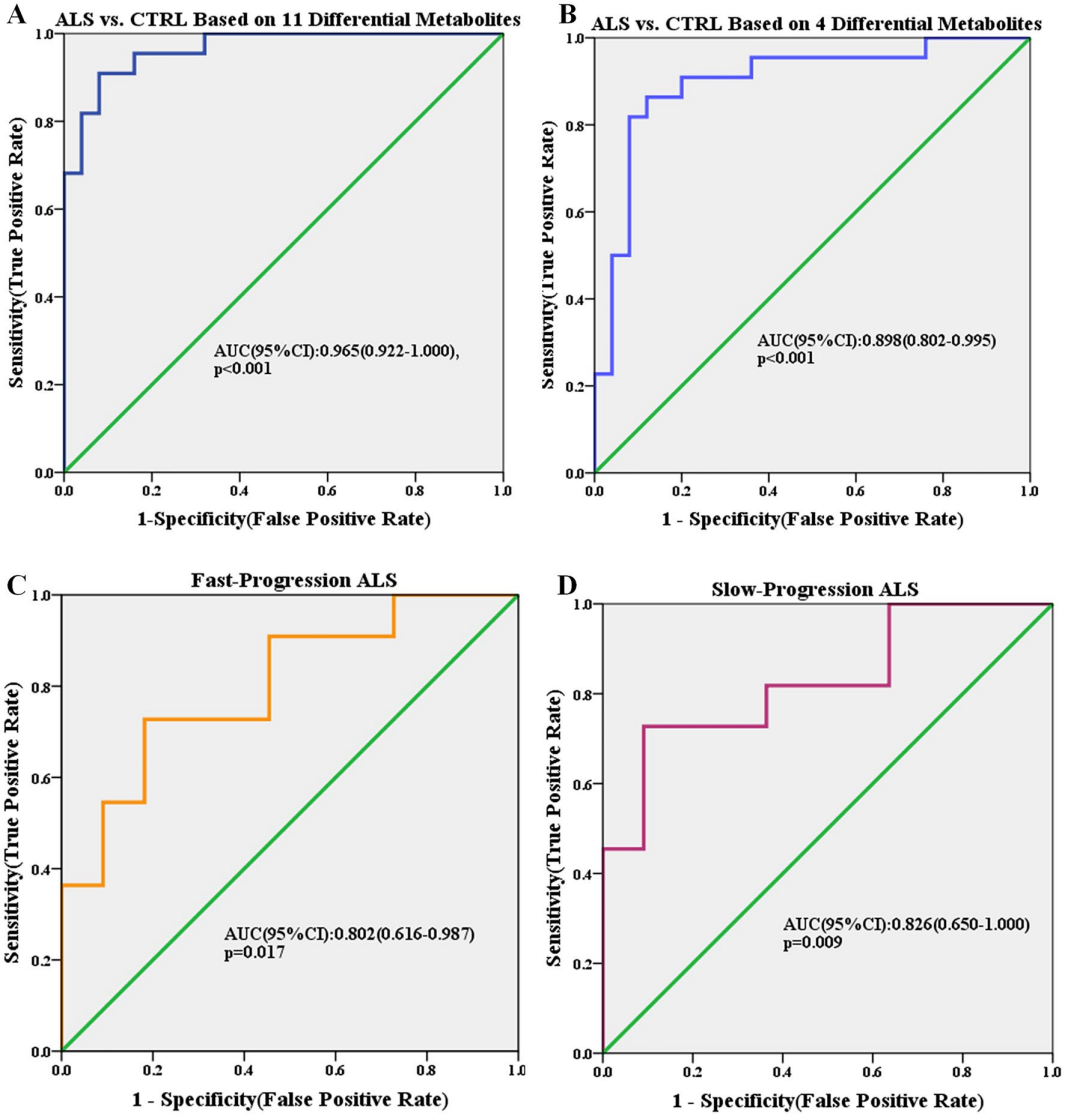
ROC curves of differential metabolites in the discrimination of ALS, fast-progression ALS, and slow-progression ALS patients.

Accurate and early diagnosis is essential for choosing appropriate treatments for ALS patients. Differential metabolites between the ALS patients and CTRL group were identified (VIP > 1, *p* < 0.05). Then logistic regression was performed and receiver operating characteristic (ROC) curves were generated. Nine differential metabolites were used to discriminate ALS patients from healthy subjects. The area under the curve (AUC) of the ROC curve was 0.952 (95% confidence interval [CI] 0.895–1.000; *p* < 0.001 (Fig. 4A). As mentioned above, 2,4,6-tri-tert-butylbenzenethiol, beta-alanine, glycine, and ethanolamine were the common differential metabolites in the ALS, fast-progression ALS, and slow-progression ALS groups. Then, these four differential metabolites were used to discriminate ALS from CTRL. ROC analysis was used to discriminate ALS patients from the CTRL group with an AUC of 0.898 (95% CI 0.802–0.995; *p* < 0.001) (Fig. 4B). Maltose, phosphoric acid, tryptophan, and pyruvic acid were identified as differential metabolites only in the fast-progression ALS group, whereas glyceric acid, beta-alanine, lactic acid, and hypoxanthine were specific differential metabolites in the slow-progression ALS group (Fig. 2D). ROC analysis showed that maltose, phosphoric acid, tryptophan, and pyruvic acid discriminated patients in the fast-progression ALS group from the CTRL group with an AUC of 0.802 (95% CI 0.616–0.987; *p* = 0.017) (Fig. 4C), whereas the slow-progression ALS and CTRL groups were separated by glyceric acid, beta-alanine, lactic acid, and hypoxanthine with an AUC of 0.826 (95% CI 0.650–1.000, *p* = 0.009) (Fig. 4D).

## Discussion

In this study, we identified differential metabolites and metabolic pathways between ALS patients and healthy subjects. Non-targeted metabolomic analysis of serum samples using GC/MS detected 75 metabolites. Multivariate analysis showed that the metabolite levels between ALS patients and the CTRL group were significantly different. Further analysis revealed that the metabolite levels and metabolic pathways were markedly altered in ALS patients with different rates of progression.

Plasma samples have been widely used to identify differential metabolites and metabolic pathways^26,27^. Cieslarova et al.^27^ measured the plasma levels of homocysteine, cysteine, methionine, and glutamic acid in ALS patients using capillary electrophoresis tandem MS, and found that glutamic acid and homocysteine might be potential biomarkers for ALS. Lawton et al.^26^ found that the plasma concentrations of 23 metabolites were significantly changed in ALS patients as well as the associated pathways including neural, hypermetabolism, oxidative damage, and mitochondrial dysfunction. Compared with plasma samples, blood has less effects on the analyte peak areas of serum specimens; therefore, serum is a better choice for metabolomic analysis^28^. In this study, we found that maltose, glyceric acid, lactic acid, beta-alanine, phosphoric acid, glutamic acid, ethanolamine, glycine, and 2,4,6-tri-tert-butylbenzenethiol were differential metabolites in ALS. All were upregulated in ALS patients, with the exception of 2,4,6-tri-tert-butylbenzenethiol.

Glutamic acid is an excitatory amino acid that is considered a key pathophysiological factor responsible for motor neuronal death in ALS^29^. Riluzole is an approved ALS drug that inhibits sodium currents and the release of glutamic acid, and prolongs the median survival of ALS patients from 11.8 to 14.8 months^1^. A study including 4571 patients and 45,411 observations with 13 months of follow-up showed that riluzole delayed the progression of ALS in patients at Milano-Torino stage 1 and King’s stages 1 and 4^9^. However, glutamatergic transmission is a complicated process involving reuptake and resynthesis of extracellular glutamate and regulation of the firing threshold of a single neuron^30^. Under physiological conditions, glutamate is released from presynaptic neurons into the synaptic cleft, while neurons and astrocytes maintain the homeostasis of glutamate^31^. When the balance is disrupted, motor neurons may die from glutamate-induced excitotoxicity^32^. In this study, we found that the serum level of glutamic acid was increased in ALS patients, suggesting that upregulated glutamic acid might induce excitotoxicity in motor neurons and ultimately lead to neuronal death. ALS patients also showed significantly altered levels of maltose, glyceric acid, lactic acid, beta-alanine, phosphoric acid, ethanolamine, glycine, and 2,4,6-tri-tert-butylbenzenethiol in the serum. The effects of these metabolites in ALS remain to be explored. Furthermore, beta-alanine metabolism, glycine serine and threonine metabolism, alanine aspartate and glutamate metabolism, pyruvate metabolism, and D-glutamine and D-glutamate metabolism were the altered metabolic pathways in patients with ALS (Fig. 2B).

It remains unknown why ALS patients show different rates of progression after onset. Meanwhile, accurately predicting ALS progression in the early stages remains a major clinical challenge. ALSFRS-r is widely used to evaluate the progression of ALS in the clinic^33^. Our results revealed that there were 10 differential metabolites in the fast-progression ALS group and another 10 differential metabolites in the slow-progression ALS group compared with the CTRL group (Table 2). By excluding the same differential metabolites in the two subgroups, we found that the levels of maltose, phosphoric acid, tryptophan acid, and pyruvic acid were significantly altered in fast-progression ALS patients, whereas glyceric acid, beta-alanine, lactic acid, and hypoxanthine were the altered differential metabolites in the slow-progression ALS group. These results suggest that ALS patients with different progression rates have differential metabolites and metabolic pathways. We further identified differential metabolic pathways in the two subgroups, and found that tryptophan metabolism was significantly changed in the fast-progression ALS group, while beta-alanine metabolism was specifically changed in the slow-progression ALS group.

Our results showed that tryptophan was downregulated and tryptophan metabolism was altered in the fast-progression ALS group. Tryptophan is one of the essential amino acids for humans and is predominately converted by intestinal microorganisms and metabolized into indole and its derivatives. Altered tryptophan metabolism is implicated in inflammatory bowel disease and irritable bowel syndrome^34^. Tryptophan and its degradation product kynurenine can pass through the highly selective blood–brain barrier, acting on neurotransmitters through glutamate receptors, and thus regulating the extracellular level of glutamate^35^. Some studies have suggested that tryptophan is associated with anxiety and depression^36,37^. Rothhammer et al.^38^ reported that metabolites derived from dietary tryptophan by gut flora activates aryl hydrocarbon receptor signaling and inhibits inflammation in the central nervous system in multiple sclerosis. Abnormal tryptophan metabolism may be related to the occurrence and development of ALS patients with fast progression. Our future studies will focus on exploring the tryptophan metabolic pathway and determining whether tryptophan dietary supplements can delay the progress of ALS.

In this study, we tried to discriminate ALS patients from the CTRL group using differential metabolites. ROC analysis revealed that ALS patients could be discriminated from CTRL by nine differential metabolites (Fig. 4A). 2,4,6-Tri-tert-butylbenzenethiol, beta-alanine, glycine, and ethanolamine are the co-differential metabolites in the ALS, fast-progression ALS, and slow-progression ALS groups. They discriminated ALS patients from the CTRL group with an AUC of 0.898 (Fig. 4B), suggesting that these metabolites might be potential diagnostic biomarkers for ALS.

In conclusion, we identified differential serum metabolites and metabolic pathways in ALS patients based on non-target GC/MS. First, we found that the metabolites in ALS versus CTRL, fast-progression ALS versus CTRL and slow-progression ALS versus CTRL were clearly discriminated. Second, ROC analysis indicated that four differential metabolites, which were identified in all ALS patients, might be potential diagnostic biomarkers for ALS. Third, ALS patients with different progression rates showed differential metabolites and metabolic pathways. Tryptophan metabolism was only changed in fast-progression ALS patients, while beta-alanine metabolism was specifically changed in the slow-progression ALS group.

Limited by the lower incidence rate of ALS and the constrains of experimental funding, our study has been carried out with a small sample. In our study, we have put on stringent inclusion criteria of ALS in order to reduce the clinical heterogeneity. In the situation of lower incidence rate and stringent inclusion criteria of ALS, our results were also credible. In the further, more studies with a larger sample size are strongly needed to validate these findings.

## Materials and methods

### Participants

Twenty-three ALS patients were recruited from the Neurology Department of the First Affiliated Hospital of Xi’an Jiaotong University (Xi’an, China) at their first diagnosis. ALS patients were strictly diagnosed by at least two experienced neurologists according to the revised El Escorial criteria. Only the ALS patients with the diagnostic grades of clinically definite (defined on clinical evidence alone by the presence of UMN and LMN signs in at least three regions) were include in the study cohort^39^.The medical records of all ALS patients were obtained including accompanying signs and symptoms, neuropathological signs, laboratory examination results including levels of low-density lipoprotein (LDL), triglyceride (TG), total cholesterol (TCHO), serum creatinine, uric acid, urea nitrogen, albumin and creatine kinase, pulmonary function test results, electromyogram (EMG) results, and the revised ALS functional rating scale (ALSFRS-r) scores. Most recruited patients were treated with riluzole or edaravone after diagnosis. To eliminate the effects of treatments on results, the rate of progression (Δr) at initial visit was calculated by dividing the ALSFRS-r total score by symptom duration (months)^40^. A control group (CTRL) consisting of 25 age- and gender-matched healthy volunteers without any nervous system diseases was also included. This study was approved by the Ethics Committee of the First Affiliated Hospital of Xi’an Jiaotong University. We confirmed that all participants had provided signed informed consent. We confirmed that this study was performed in accordance with relevant guidelines and regulations.

### Serum sample collection

Peripheral venous blood (3 mL) was obtained from all participants in the morning and then centrifuged at 3000 rpm for 15 min. A volume of 0.3 mL supernatant was collected and stored at − 80 °C until use. Each serum sample (100 µL) was thawed at 4 °C, transferred into a 1.5 mL centrifuge tube, and then added to 400 µL pre-chilled methanol. After 1 min of vortex mixing, 60 µL of 2-chloro-L phenylalanine (0.2 mg/mL stock in methanol) and 60 µL heptadecanoic acid (0.2 mg/mL stock solution) were added to each sample as internal quantitative standards, and vortexed for an additional 1 min. The mixed-serum samples were centrifuged for 10 min at 12,000 rpm at 4 °C, and then the supernatant was transferred to a 1.5 mL centrifuge tube. Samples were vacuum dried, followed by treatment with 60 µL of 15 mg/mL methoxyamine pyridine solution for 120 min at 37 °C. Then, 60 µL BSTFA reagent (containing 1% TMCS) was added to each sample and incubated for 90 min at 37 °C. Finally, the mixture was centrifuged at 12,000 rpm for 10 min at 4 °C, and the supernatant was collected.

### GC/MS metabolomic assay, quality control, and extraction

Gas chromatography (Agilent 7890A; Agilent Technologies, Santa Clara, CA, USA) was performed on an HP-5 ms GC column (5% phenyl/95% methylpolysiloxane, 30 m × 250 µm i.d., 0.25 µm film thickness; Agilent J & W Scientific, Folsom, CA, USA) at a constant flow rate of 1 mL/min helium. A volume of 1 µL sample was injected in split mode (split ratio 20:1) using an autosampler. The injection temperature was 280 °C. The interface was set to 150 °C and the ion source was adjusted to 230 °C. In this program, the temperature was initially at 60 °C (2 min), increased to 300 at a rate of 10 °C /min, and maintained at 300 °C for 5 min. MS (Agilent 5975C) was performed using the full-scan method from 35 to 750 (m/z). For quality control (QC), 20 µL of each sample was extracted and mixed. These QC samples were used to monitor deviations of the analytical results from the pool mixtures, and compare them to the errors caused by the analytical instrument itself. The remaining samples were analyzed by GC/MS. The raw GC/MS data were converted to the netCDF format (XCMS input file format)^41^ using G1701 MSD ChemStation software (E. 02.00.493). The XCMS package in R software (v3.3.2) was used for peak identification, peak filtration, and peak alignment. The parameters were as follows: fwhm = 3, snthresh = 0, mzdiff = 0.5, profmethod = “binlin”, bw = 2, minfrac = 0.3. A data matrix including the mass-to-charge ratio (m/z), retention time, and intensity was generated. Metabolites were characterized by AMDIS software according to the National Institute of Standards and Technology, Wiley Registry metabolomics database, and the Golm Metabolome Database, and were then confirmed by standards.

### Statistical analysis

Data are expressed as the mean ± standard deviation (SD) for continuous variables and as a number (percentage) for categorical variables. The difference in age, body mass index (BMI), and laboratory examination results between ALS patients and CTRL were analyzed by the Student’s *t*-test using SPSS version 20.0 (IBM, Armonk, NY, USA). Before analysis, metabolomic data were normalized and scaled to unit variance. Multivariate data analysis was performed using SIMCA-P (v13.0) and R software. The orthogonal projections to latent structures discriminant analysis (OPLS-DA) model was used to characterize serum metabolic disturbance. The Student’s *t*-test was used to compare each metabolite between the ALS and CTRL groups. The AVONVA analysis was used to compare each metabolite among ALS, CTRL, Fast-progression ALS and Slow-progression ALS. The heatmap and hierarchical clustering were generated using the pheatmap package in R software (v3.3.2). Kyoto Encyclopedia of Genes and Genomes and MetaboAnalyst were used to identify metabolic pathways. The sensitivity and specificity of the diagnosis and progression of ALS with different groups of differential metabolites were analysis by multivariate ROC curve analysis in SPSS: different combinations of metabolites were performed by Binary logistic regression analysis, and then the probabilities value was got; the obtained probabilities value was used as the test variables to carry out the ROC curve analysis. *p* < 0.05 was considered statistically significant.

## Data Availability

The data that supported our new findings would be acquired after author’s permission.
